# Einsatz eines digitalen Gesundheitslotsen in der Notaufnahme: Erfahrungen und Akzeptanz bei Patient:innen und Ärzt:innen

**DOI:** 10.1007/s00103-026-04302-x

**Published:** 2026-09-11

**Authors:** Maria Schwenke, Pascal Samtlebe, Susann Homann, Anja Knöchelmann

**Affiliations:** 1 Institut für Medizinische Soziologie, Medizinische Fakultät, Martin-Luther-Universität Halle-Wittenberg, Universitätsmedizin Halle, Magdeburger Straße 8, 06112 Halle (Saale), Deutschland; 2 Digital Transformation Department, Halle University Hospital, Halle (Saale), Sachsen-Anhalt Deutschland

**Keywords:** Symptomchecker, Technologieakzeptanz, Patientensteuerung, Digitale Selbsteinschätzung, Triage, Symptom checker, Technology acceptance, Patient navigation, Digital self-assessment, Triage

## Abstract

**Hintergrund:**

Die zunehmende Inanspruchnahme zentraler Notaufnahmen (ZNA) durch niedrigkomplexe Fälle stellt Krankenhäuser vor Herausforderungen. Seit 2023 wird in der ZNA des Universitätsklinikums Halle (Saale) ein digitaler Gesundheitslotse (DGL) eingesetzt. Das KI-basierte System ermöglicht mittels symptombasierter Selbstanamnese eine Ersteinschätzung und empfiehlt Versorgungsstrukturen. Untersucht wurden Nutzungserfahrungen und Bewertung durch Patient:innen sowie Akzeptanz und Nutzung der DGL-Ergebnisse durch Ärzt:innen.

**Methoden:**

In einer explorativen Studie mit konvergentem Mixed-Methods-Design wurden 82 Patient:innen der Manchester-Triagestufen 4 oder 5 zu Nutzungserfahrungen und digitaler Gesundheitskompetenz mittels der Kurzform des „User Experience Questionnaire“ (UEQ‑)S und der „eHealth Literacy Scale“ (eHEALS) befragt (08/2025–01/2026) und 8 Interviews mit Ärzt:innen inhaltsanalytisch ausgewertet.

**Ergebnisse:**

Beide Gruppen standen dem DGL überwiegend offen gegenüber. Patient:innen bewerteten Verständlichkeit (61 %), Design (54 %) und Datensicherheit (52 %) überwiegend positiv, bemängelten jedoch teils die Symptomerfassung. Instabile Internetverbindungen und fehlende technische Ausstattung erschwerten die Nutzung. Ärzt:innen äußerten Bedenken hinsichtlich Validität und Verlässlichkeit. Eine feste Integration in die Arbeitsabläufe bestand nicht. Potenziale sahen Patient:innen in der ersten Orientierung, Ärzt:innen in der (Nach‑)Triage und Entscheidungen über Wartezeiten, in Informationsstrukturierung und der Überwindung von Sprachbarrieren. Zeitdruck und gesundheitliche Belastung der Patient:innen limitieren die Anwendung in der ZNA.

**Diskussion:**

Entscheidend für die Implementierung ist weniger die individuelle Akzeptanz als die systemische Passung. Erforderlich sind der Abbau technischer Hürden, bessere Information, flexiblere Datenerhebung und die Integration in klinische Abläufe. Dies sollte Gegenstand zukünftiger Forschung sein.

## Abstract (en)

**Background:**

The increasing use of emergency departments (EDs) for low-acuity cases leads to growing challenges for hospitals. Since 2023, a digital patient navigator (DGL) has been implemented in the ED of the University Hospital Halle (Saale). The AI-based decision support system provides an initial assessment based on a symptom-based self-anamnesis and recommends appropriate care pathways; the collected data are also available to medical staff for consultation. This study examines patients’ user experiences and evaluations as well as physicians’ acceptance and use of the DGL results.

**Methods:**

In an exploratory convergent mixed-methods study, 82 patients classified in Manchester Triage Levels 4 or 5 were surveyed using the UEQ‑S and eHEALS instruments (August 2025 to January 2026). A further eight physicians were interviewed and analyzed using structured qualitative content analysis.

**Results:**

Both groups were generally open to the usage of the DGL. Patients rated comprehensibility (61%), design (54%), and data security (52%) mostly positively, while the symptom assessment was criticized. Unstable internet connections and a lack of technical equipment made usage difficult. Physicians expressed concerns regarding validity and reliability. An integration into workflows was not established. Patients saw potential in the initial orientation, while physicians saw potentials in (post-)triage and decisions regarding waiting times, information structuring, and overcoming language barriers. Time constraints and the patients’ health limitations and impairments limited the use in the ED.

**Discussion:**

Critical to the implementation of the DGL was systemic fitting rather than individual acceptance. Technical barriers need to be addressed; better information and flexible data collection were deficits that hindered the sustained integration into clinical workflows. This should be the focus of future research.

## Einleitung

Die stationäre Krankenversorgung in Deutschland steht unter strukturellem Druck. Finanzielle Defizite sorgten langfristig für Stagnationen der Personalausstattung und Investitionsstau in Krankenhäusern. Besonders stark betroffen ist die Notfallversorgung [1–4].

Für Patient:innen bieten Notaufnahmen eine unmittelbare Versorgungsmöglichkeit unabhängig von der Tageszeit und fungieren zunehmend als Alternative bei eingeschränkter Verfügbarkeit ambulanter Versorgungsangebote, obwohl eine Behandlung in der Notaufnahme in vielen Fällen weder zeitkritisch noch zwingend erforderlich ist. In der Folge steigen die personellen und finanziellen Bedarfe, wodurch sich die wirtschaftliche Lage der Krankenhäuser verschärft. Daher sind innovative Lösungen notwendig, um die Qualität der Versorgung und Behandlung sicherzustellen [5–8].

Digitale Unterstützungssysteme werden zunehmend als Instrumente zur Optimierung der medizinischen Versorgung diskutiert und erprobt. Mit dem Krankenhauszukunftsgesetz wurde im Jahr 2020 das Ziel der Förderung einer umfassenden technischen Modernisierung verfolgt, mit dem Fokus auf Notfallversorgung und patient:innenorientierte digitale Angebote. Auf dieser Grundlage wurde ein „digitaler Gesundheitslotse“ (DGL) am Universitätsklinikum Halle (Saale) (UKH) in Sachsen-Anhalt eingeführt.

Der DGL ist ein Symptomchecker mit Steuerungsfunktion, in dem die Patient:innen im Rahmen einer strukturierten Eigenanamnese ihre Symptome selbstständig im Chatformat eingeben und auf dieser Basis Empfehlungen für eine angemessene medizinische Versorgung erhalten, die anschließend ärztlicherseits berücksichtigt werden können. Grundlage ist das als Medizinprodukt zugelassene, KI-basierte Swiss Medical Assessment System (SMASS, in4medicine AG, Bern, Schweiz). Die Anwendung erfüllt die regulatorischen Anforderungen der Medizinprodukteverordnung und basiert auf einem evidenzbasierten Regelwerk mit medizinischen Warnzeichen (Red Flags). SMASS wurde in Deutschland für verschiedene Einsatzbereiche der Akut- und Notfallversorgung adaptiert und unter anderem seit 2018 als „Strukturierte medizinische Ersteinschätzung in Deutschland“ (SmED) im Rahmen der 116117 eingesetzt [9]. Am UKH wurde der DGL an die Versorgung durch die Zentrale Notaufnahme (ZNA) angeschlossen. Der DGL kann sowohl präklinisch als auch in der ZNA eigenständig auf persönlichen Endgeräten genutzt werden, wobei bei Bedarf die generierten Daten in das Krankenhausinformationssystem (KIS) übernommen werden.

**Abb. 1:**
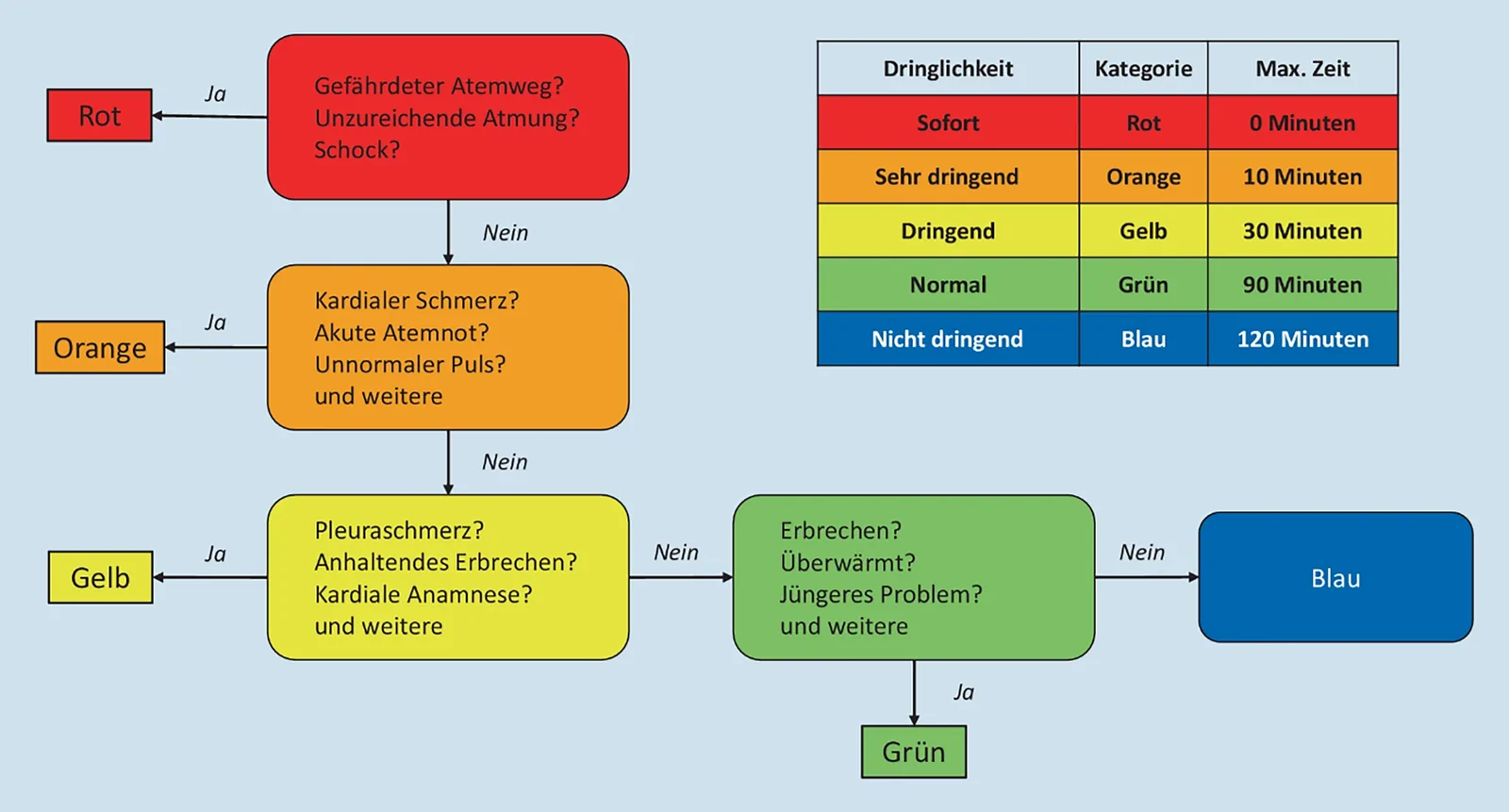
Schematische Darstellung des Manchester-Triage-System (MTS) mit 5 Dringlichkeitsstufen und Wartezeiten (Mayerhofer et al. [10])

In der ZNA des UKH wird ausgewählten Patient:innen mit niedriger Behandlungsdringlichkeit (Manchester-Triage-System (MTS), Stufen 4 oder 5) aktiv die Nutzung des DGL empfohlen. Die dabei erhobenen Daten werden anschließend automatisch im KIS bereitgestellt. Das MTS dient der Einschätzung der Dringlichkeit in der medizinischen Versorgung und ordnet Patient:innen anhand von Leitsymptomen 5 Dringlichkeitsstufen zu, die zugleich die maximale Wartezeit der Patient:innen bestimmen (Abb. 1; [10]).

Die erfolgreiche Integration des DGL hängt entscheidend von den Nutzungserfahrungen und der Akzeptanz aller Anwender:innen ab. Die begleitende evaluative Forschung widmete sich daher der Frage, wie der DGL aus Sicht der Patient:innen und Ärzt:innen wahrgenommen, genutzt und bewertet wird.

## Methodik

Für diese explorative Studie wurde ein multiperspektivisches konvergentes Mixed-Methods-Design gewählt. Die quantitative Befragung erfasste die Nutzungserfahrung aus Patient:innensicht. Die qualitative Studie untersuchte Dimensionen der Technologieakzeptanz im ärztlichen Kontext. Die Befragungen wurden unabhängig voneinander durchgeführt und analysiert. Die Ergebnisse wurden anschließend gemeinsam interpretiert und zueinander in Beziehung gesetzt.

### Quantitative Befragung

Die Onlinebefragung zum DGL erfolgte über Lime-Survey (LimeSurvey GmbH, Hamburg, Deutschland) von August 2025 bis Januar 2026. Eingeschlossen wurden Personen ≥ 18 Jahre, die in der ZNA des UKH vorstellig wurden, im Rahmen der regulären Triage den MTS-Stufen 4 oder 5 zugeordnet wurden und vom Pflegepersonal eine Empfehlung zur Nutzung des DGL sowie Informationen zur Teilnahme an der Onlinebefragung in Form eines Handouts erhielten. Die Empfehlung erfolgte in Abhängigkeit von den verfügbaren personellen und zeitlichen Ressourcen und nach Einschätzung des Fachpersonals hinsichtlich sprachlicher oder gesundheitlicher Einschränkungen. Die Befragung wurde in deutscher Sprache durchgeführt. Auf Grundlage der Selbstangaben zur Durchführung und zum Abschluss des DGL wurden die Teilnehmenden in Nutzer:innen und Nichtnutzer:innen eingeteilt.

#### Variablen.

Erhoben wurden soziodemografische Angaben (Alter, Geschlecht, höchster Bildungsstand), Informationen zu Bekanntheitsdauer des DGL, bisheriger Nutzungshäufigkeit, ursprüngliche Informationsquellen zum DGL sowie zur beabsichtigten zukünftigen Nutzung und Weiterempfehlung. Bei Ablehnung des DGL wurden die Gründe abgefragt sowie 6 offene Fragen zu wahrgenommenen Barrieren und potenziellen Motivationsfaktoren gestellt.

Personen, die den DGL nutzten, beantworteten Fragen zu ihren Nutzungserfahrungen anhand der Kurzform des „User Experience Questionnaire“ (UEQ-S), die 8 Gegensatzpaare in den 2 Dimensionen pragmatische und hedonische Qualität umfassen (Bewertung: neutral = −0,8 bis < 0,8; positiv ≥ 0,8; negativ ≤ −0,8; [11]). Ergänzend wurden Aspekte der Nutzungsfreundlichkeit, Nützlichkeit und Informiertheit erhoben. Positive und negative Aspekte der Anwendung wurden mittels offener Fragen erfasst*.*

Die digitale Gesundheitskompetenz wurde mit der eHealth Literacy Scale (eHEALS) erhoben [12]. Der Gesamtscore wurde durch Summation der Items gebildet (8–40 Punkte), wobei höhere Werte eine höhere selbst eingeschätzte digitale Gesundheitskompetenz anzeigen.

#### Datenauswertung.

Die statistische Auswertung erfolgte deskriptiv. Gruppenunterschiede wurden mittels t‑Tests (metrische Daten) und Chi-Quadrat-Tests bzw. Exact-Fisher-Test (kategoriale Daten) analysiert. Unterschiede im eHEALS-Score wurden unter der Annahme untersucht, dass Nutzer:innen über eine höhere digitale Gesundheitskompetenz als Nichtnutzer:innen verfügen. Die offenen Angaben wurden mittels qualitativer Inhaltsanalyse systematisch codiert und zusammengefasst.

### Qualitative Befragung

Die qualitative Befragung des ärztlichen Personals folgte einem explorativen Ansatz. Von Juni bis September 2025 wurden leitfadengestützte, semistrukturierte Interviews mit 8 Ärzt:innen durchgeführt, die zu dem Zeitpunkt der Interviews in der ZNA des UKH tätig waren, unabhängig von Beschäftigungsdauer und Erfahrungsniveau. Die Interviews wurden aufgezeichnet, vollständig transkribiert und anonymisiert.

Der Leitfaden erfasste sowohl nutzungsbezogene als auch organisatorische Aspekte und orientierte sich am Technologiakzeptanz-Modell der „Unified Theory of Acceptance and Use of Technology“ (UTAUT; [13]). Zentrale Befragungsgegenstände waren die Bewertung der DGL-Ergebnisse (PDF-Dokumente) sowie deren Integration in die digitale Infrastruktur und die Arbeitsabläufe der ZNA.

Die Auswertung erfolgte mittels inhaltlich strukturierender qualitativer Inhaltsanalyse mit MAXQDA26 [14] anhand eines deduktiv entwickelten und induktiv ergänzten Kategoriensystems.

## Ergebnisse

### Beschreibung der Stichproben

Das Sample der qualitativen Interviews umfasste 2 Oberärzt:innen sowie 6 Assistenzärzt:innen unterschiedlicher Fachrichtungen (Chirurgie, Traumatologie, innere Medizin, Allgemeinmedizin, Anästhesie) mit einer Beschäftigungsdauer zwischen wenigen Monaten bis zu mehreren Jahren.

**Abb. 2:**
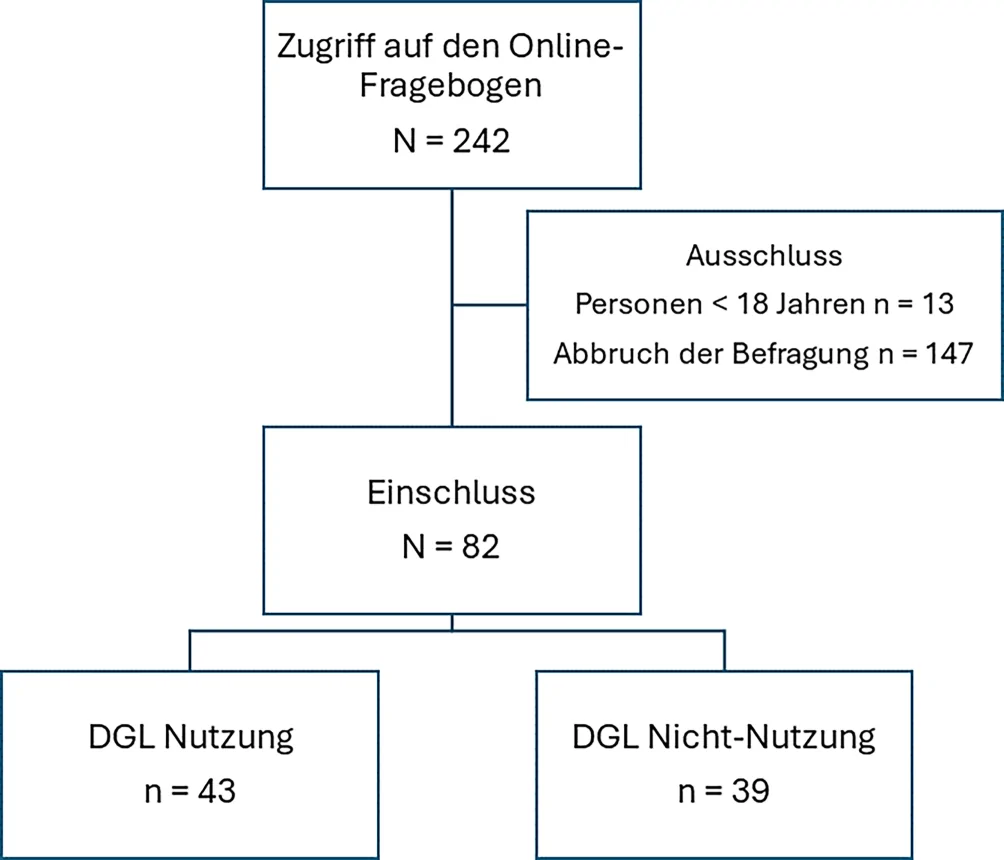
Einschluss an der Befragung teilnehmender Patient:innen in die Auswertung. *DGL* digitaler Gesundheitslotse

In dem 6‑monatigen Befragungszeitraum (08/2025–01/2026) wurden in der ZNA ca. 15.000 Personen mit MTS 4 oder 5 vorstellig. Erfahrungswerte aus dem Regelbetrieb zeigen, dass 12 % der Patient:innen (MTS 4/5) den DGL nutzen. Der Fragebogen wurde insgesamt von 242 Personen aufgerufen und von 82 Personen, die die Einschlusskriterien erfüllten, vollständig ausgefüllt. Davon hatten 43 den DGL genutzt, während 39 die Anwendung nicht genutzt hatten (Abb. 2). Die Geschlechterverteilung war nahezu ausgeglichen; das Durchschnittsalter betrug 41 Jahre (Spannweite: 18–92 Jahre; Tab. 1). Die Mehrheit hatte als höchsten Abschluss einen schulischen Abschluss (60 %). Signifikante demografische Unterschiede zwischen den Gruppen ließen sich nicht beobachten, wenngleich der Anteil von Personen mit akademischem Abschluss in der Nutzungsgruppe höher war (30 % vs. 10 %; *p* = 0,054). Unterschiede zeigten sich hingegen bei der digitalen Gesundheitskompetenz: Nutzer:innen wiesen einen leicht, aber signifikant höheren eHEALS-Score auf als Nichtnutzer:innen (Score 29,3 vs. 26,1 Punkte; *p* = 0,028).

**Tab. 1:** Stichprobenbeschreibung der teilnehmenden Patient:innen (*N* = 82) nach Geschlecht, Alter, Bildung, Gesundheitskompetenz und Nutzung des digitalen Gesundheitslotsen (DGL)

Variable	Gesamt *N* = 82	Nutzung *n* = 43	Nichtnutzung *n* = 39
*Geschlecht, n* (%)			
Weiblich	40 (48,8)	21 (48,8)	19 (48,7)
Männlich	42 (51,2)	22 (51,2)	20 (51,3)
*Alter*, Jahre, M (SD)	41,1 (16,5)	41,7 (16,2)	40,5 (17,0)
*Bildung*, höchster Abschluss, *n* (%)			
Schulisch	49 (59,8)	21 (48,8)	28 (71,8)
Beruflich	14 (17,1)	9 (20,9)	5 (12,8)
Akademisch	16 (19,5)	12 (27,9)	4 (10,3)
Sonstige/k. A.	3 (3,7)	1 (2,3)	2 (5,1)
*Digitale Gesundheitskompetenz eHEALS,* M (SD)	27,8 (6,8)	29,3 (6,7)	26,1 (6,6)

*k.A.* keine Angabe, *M* Mittelwert, *SD* Standardabweichung

Der überwiegende Teil aller befragten Patient:innen hatte die Anwendung erst kürzlich kennengelernt (92 %) und sie höchstens einmal durchgeführt (96 %). Der Erstkontakt erfolgte überwiegend in der ZNA durch Empfehlung des Pflegepersonals (70 %), selten über andere Informationsquellen wie die Website des Klinikums (15 %), soziale Kontakte oder Werbung (6 %).

### Nutzungserfahrungen der Patient:innen

Patient:innen bewerteten die Anwendung hinsichtlich der Verständlichkeit (61 %), des Designs (54 %) und der Datensicherheit (52 %) als überwiegend benutzungsfreundlich (Tab. 2). Die Einschätzungen zur Nützlichkeit der Informationen und zum Vertrauen in die Empfehlungen des DGL fielen überwiegend neutral aus; zugleich zeigte sich ein hoher Anteil fehlender Angaben (30 % bzw. 26 %).

**Tab. 2:** Benutzungsfreundlichkeit und Nützlichkeit des digitalen Gesundheitslotsen (DGL) aus Patient:innenperspektive (*n* = 43)

*Items zu Benutzungsfreundlichkeit und Nützlichkeit*	Stimme überhaupt nicht zu *n* (%)	Stimme eher nicht zu *n* (%)	Teils, teils *n* (%)	Stimme eher zu *n* (%)	Stimme sehr zu *n* (%)	Keine Angabe *n* (%)
Ich habe die Fragen des DGL gut verstanden	2 (4,7)	0 (0)	7 (16,3)	10 (23,3)	16 (37,2)	8 (18,6)
Ich empfand das Design des DGL als ansprechend	2 (4,7)	0 (0)	10(23,3)	14 (32,6)	9 (20,9)	8 (18,6)
Ich fühlte mich bei der Benutzung des DGL hinsichtlich meiner Daten sicher	1 (2,3)	0 (0)	13 (30,2)	9 (20,9)	13 (30,6)	7 (16,3)
Der DGL hat mir nützliche Informationen zu meinem Gesundheitsproblem gegeben	2 (4,7)	6 (14,0)	11 (25,6)	9 (20,9)	2 (4,7)	13 (30,2)
Ich vertraue den Empfehlungen des DGL	3 (7,0)	2 (4,7)	14 (32,6)	10 (23,3)	3 (7,0)	11 (25,6)

Im UEQ‑S zeigte sich im Mittel eine positive Bewertung der pragmatischen (M = 0,93) und eine neutrale Bewertung der hedonischen Qualität (M = 0,70). Insbesondere die Leichtigkeit der Bedienung (M = 1,3), die Neuartigkeit (M = 1,2) und die Übersichtlichkeit der Anwendung (M = 1,0) wurden positiv bewertet. Der Gesamtwert des UEQ‑S fiel neutral aus (M = 0,73).

**Abb. 3:**
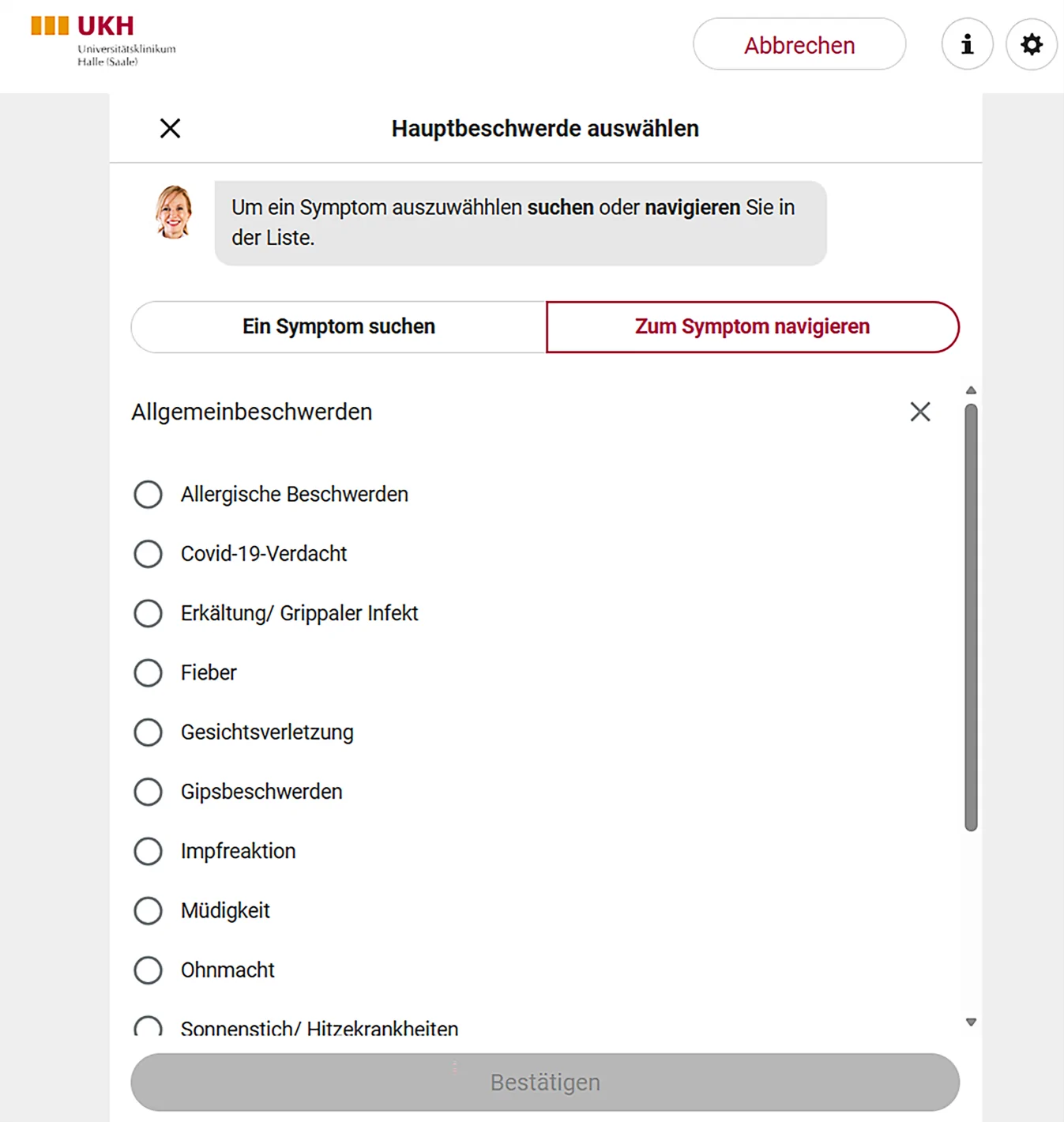
Beispiel der Benutzeroberfläche zur Symptomeingabe des digitalen Gesundheitslotsen (DGL, Swiss Medical Assessment System (SMASS); technisches Release 5.4.10; medizinisches Release 9.6)

Neben der Empfehlung in der ZNA (40 von 43 Nutzer:innen) wurde der DGL zur Einschätzung der gesundheitlichen Situation einer Begleitperson, zum Verständnis der eigenen Symptome sowie zur Einschätzung der Dringlichkeit und des geeigneten Behandlungsortes genutzt. Kritisch wurde die Symptomabfrage (Abb. 3) bewertet, da die vorgegebenen Antwortoptionen die individuellen Beschwerden nicht hinreichend abbildeten. Insbesondere das Fehlen einer Freitexteingabe sowie die eingeschränkte Möglichkeit, den Ort der Beschwerden oder das betroffene Organ präzise anzugeben, führte zu als unpassend empfundenen Folgefragen und wenig zielgerichteten Ergebnissen.

13 von 39 der Nichtnutzer:innen gaben technische Probleme wie eine Fehlfunktion des Zugangslinks bzw. der Internetverbindung und mangelnde technische Ausstattung an. Weitere Gründe waren fehlende Kenntnis oder fehlendes Interesse (*n* = 14), fehlende Zeit (*n* = 6) und unzureichende Informationen oder Zweifel an der Zuverlässigkeit (*n* = 5).

Hinsichtlich der künftigen Nutzungsabsichten äußerten sich 35 % aller Befragten positiv, während 16 % eine weitere Anwendung ablehnten und der größte Teil der Befragten (46 %) unentschlossen blieb. 16 % der Nutzer:innen und 41 % der Nichtnutzer:innen würden die Anwendung weiterempfehlen (*p* = 0,043).

### Einstellungen und Nutzungserfahrungen der Ärzt:innen

#### Allgemeine Einschätzung

Der überwiegende Teil der Ärzt:innen äußerte sich grundsätzlich positiv gegenüber dem Einsatz von digitalen Anwendungen wie Symptomcheckern in der ZNA. Dabei wurden die potenziellen Vorteile maßgeblich aufseiten der Patient:innen formuliert, der Nutzen für die ärztliche Arbeit selbst wurde weitaus kritischer eingeschätzt, wie folgende Aussage verdeutlicht:

„Ich finde das generell eigentlich eine gute Idee, einfach um den Leuten schon so ein bisschen ein Gefühl zu geben, wie notfällig sie selbst sind, weil das ist ja häufig so ein bisschen, manchmal falsch eingeschätzt. …, zum anderen ist es natürlich für uns auch halt so ein bisschen als Hilfestellung und Orientierung schon mal gedacht“ (Ärzt:in 2).

Ebenso wurden grundsätzliche Bedenken hinsichtlich der Zuverlässigkeit der Technik und der Patient:innenangaben geäußert.

Trotz allgemein positiver Einstellungen fällt die tatsächliche Nutzung des DGL gering aus. Die Hälfte der befragten Ärzt:innen gab an, die DGL-Ergebnisse im Rahmen einer Konsultation angesehen zu haben; für keine der befragten Personen oder deren kollegiales Umfeld sei die Nutzung jedoch ein fester Bestandteil der Arbeit.

#### Hindernisse

##### Informiertheit.

Ein Grund für die Nichtnutzung waren mangelnde Informationen zur Funktionsweise und Implementierung des DGL in der ZNA. Schulungen wurden nur vereinzelt erwähnt und unterschiedlich bewertet. Zudem bleibt angesichts der hohen Arbeitsdichte nur wenig Raum für zusätzliche Neuerungen und das gut funktionierende Triage-System wird als ausreichend wahrgenommen.

##### Technische und informative Zugänglichkeit.

Vor allem die schwierige Auffindbarkeit des DGL-Dokuments im KIS, was auf eine Verzögerung bei der Übertragung zurückgeführt wurde, sowie der mehrschrittige Zugang verhinderten eine kontinuierliche Einbindung in die zeitlich angespannten Arbeitsroutinen. Die informative Qualität der DGL-Ergebnisse wurde gemischt bewertet. Zwei der befragten Ärzt:innen beschrieben das Dokument als übersichtlich und die umfassenden Differenzialdiagnosen als hilfreich. Weitere Befragte beschrieben es als zu umfang- und textreich, informativ überfordernd und angesichts der verfügbaren Triagierung als redundant.

##### Symptomeingabe.

Die Ärzt:innen kritisierten die standardisierte Erfassung der Symptome und klinischen Entscheidungsprozesse im DGL und bezweifeln, dass diese der medizinischen Individualität und Komplexität gerecht werden:

„Diese Tools wollen immer ärztliche Entscheidungen operationalisieren, aber irgendwie funktioniert’s so nicht so ganz. Also ich arbeite nicht immer nach irgend so einem komischen Schema die Sachen ab, sondern da ist eben viel Bauchgefühl oder wie man es nennen soll oder klinische Erfahrung“ (Ä5).

Zusätzlich wurden Bedenken hinsichtlich der Kompetenzen der Patient:innen zur zuverlässigen Angabe geäußert. Die Selbsteinschätzung von Symptomart und -intensität erfordere ein Maß an Gesundheitskompetenz, das nicht bei allen Nutzenden, insbesondere in Notfallsituationen, vorausgesetzt werden könne. Ärzt:innen nahmen eine Tendenz zur bewussten Dramatisierung der Symptome durch Patient:innen an, um den Besuch der ZNA zu legitimieren.

„Die Patienten, die hierherkommen, die werden natürlich auch die stärksten Symptome mit angeben, weil sie wollen ja auch quasi rechtfertigen, dass sie in der Notaufnahme sitzen. Und dann beim weiteren Anamnesegespräch stellt sich heraus naja, es ist ja eigentlich gar nicht so akut, sondern es sind schon acht Wochen her“ (Ä4).

Zudem beobachteten mehrere Ärzt:innen die Tendenz des DGL zum Upcoding: Bei Grenzfällen tendiere der Algorithmus systematisch zu einer Empfehlung des Notaufnahmebesuchs. Dies kann potenziell das dahinterliegende Ziel einer Patient:innensteuerung aus der Akutversorgung heraus und somit einer Verringerung der Inanspruchnahme der Notaufnahme konterkarieren. Einen Grund dafür suchte ein Arzt in der Notwendigkeit zur rechtlichen Absicherung des DGL selbst:

„Um sicherzustellen, dass man da keine Sachen übersieht, müssen die ja immer so programmiert sein, dass sie extrem vorsichtig sind und im Zweifel sowieso die Leute schicken. Und die Leute kommen ja so auch schon mit Bagatellen und deswegen wäre ich mir da eher unsicher, dass das ein wirklicher Zugewinn ist. Also das sind rechtliche Probleme … Deswegen sehe ich, also sehe ich das eher kritisch“ (Ä5).

#### Patient:innenkontakt

Auf die Behandlung oder das Gespräch mit Patient:innen hatte der DGL nur geringen Einfluss, jedoch äußerten nahezu alle Ärzt:innen sowohl Potenziale als auch Bedenken. Patient:innen, die vorab den DGL genutzt hatten, wirkten mitunter „besser sortiert“ (Ä6) und durch die Vorbereitung komme „man dann mit den Leuten besser gleich zum Ziel“ (Ä1). Das multilinguale Angebot des DGL überbrückt Sprachbarrieren und erhöht die wahrgenommene Zuverlässigkeit dieser Angaben. Zeit- oder Effizienzgewinne wurden nicht wahrgenommen.

Eine Beeinflussung des Gesprächs durch das Vorhandensein des DGL-Dokuments wird nicht gesehen. Vielmehr wurden die darin enthaltenen Angaben im direkten Kontakt erneut besprochen. Darüber hinaus legten mehrere Ärzt:innen Wert auf eine persönliche Anamnese, einerseits um den Leidensdruck anzuerkennen und zwischenmenschlichen Kontakt herzustellen, andererseits um dem eigenen Anspruch an eine aufmerksame Zuwendung gegenüber den Patient:innen gerecht zu werden:

„Jetzt kommt der Patient rein, ich nehme mir jetzt halt zehn Minuten für den und anamnestiziere den auch selbst und meistens reicht das ja auch für eine gute Analyse. Während ich hier [für das DGL-Dokument] … wahrscheinlich schon zwei Minuten brauche, um mir das adäquat durchzulesen“ (Ä7).

Ärzt:innen befürchten zudem Fixierungsfehler im Sinne eines Automation-Bias (Verzerrung zugunsten automatisierter Empfehlungen). Die durch das Tool vorgegebene Diagnose trete ihnen als technisch erzeugt und scheinbar validiert entgegen. Durch die Angabe einer errechneten Wahrscheinlichkeit bestehe das Risiko, sich zu stark auf das DGL-Ergebnis zu fixieren und dadurch andere diagnostische Möglichkeiten weniger zu berücksichtigen. Gerade für Berufseinsteigende werde dies als Risiko gesehen und empfohlen, die DGL-Ergebnisse daher erst nach der ärztlichen Einschätzung hinzuzuziehen.

#### Potenziale zur Nutzung und Verbesserung

Die Ärzt:innen sahen Nutzungspotenziale in der Möglichkeit, den DGL als zusätzliche Informationsquelle für die Organisation des Wartezimmers der ZNA heranzuziehen. Das DGL-Ergebnis könne bereits vor dem Patient:innenkontakt zusätzliche Einblicke in den Behandlungsbedarf geben und dabei helfen einzuschätzen, „wenn jetzt jemand sehr lange wartet, ob das in Ordnung ist oder nicht“ (Ä2). Zudem könnte der DGL einen Überblick über die wartenden Personen erleichtern und bei längeren Wartezeiten von Patient:innen mit geringer Behandlungskomplexität eine zusätzliche Absicherung in Form einer Nachtriagierung ermöglichen:

„Also wie so eine Nachtriagierung … für den häufigen Fall, dass man einfach nicht alle Patienten zeitgemäß sehen kann. Und dann quasi nochmal filtern muss, okay, dann kommt aber doch die beiden Personen vor der einen Person dran oder so. … aber dass man dann vielleicht die Leute, die dann hochakut was haben, dass man die tatsächlich wirklich schnell einsammeln kann, auch wenn eine Flut an Patienten da ist“ (Ä3).

Weiterhin könnten die DGL-Ergebnisse bei der Triage eine schnellere und korrekte Zuweisung zum passenden fachärztlichen Bereich unterstützen. Der DGL könne damit zu einer besseren Strukturierung interner Abläufe und der Organisation beitragen. Das Potenzial zur direkten Steuerung der Patient:innen aus der ZNA in andere Versorgungsstrukturen werde hingegen angezweifelt.

Verbesserungspotenziale bestehen in einer übersichtlicheren Aufarbeitung der DGL-Ergebnisse durch farbliche Markierung relevanter Inhalte und Reduktion unauffälliger und irrelevanter Ergebnisse. Zudem sollten die Ergebnisse im KIS leichter auffindbar sein, z. B. über ein eigenes optisches Symbol in der Patient:innenübersicht oder Schnellzugriff im Triagierungsbereich.

Aus Sicht der Ärzt:innen bietet der DGL Potenzial für Effizienzgewinne, wenn eine strukturierte Übernahme der Daten ins KIS erfolgt. Erst durch eine solche Integration sowie die damit verbundene Reduktion von Dokumentationslast und Mehrfacheingaben entstünden Anreize zur Nutzung. Dabei wurde auf Möglichkeiten hingewiesen, die Datenerfassung um Aspekte der Sozialanamnese, den Pflegegrad oder das Einlesen des bundeseinheitlichen Medikationsplans zu erweitern:

„Also meiner Meinung nach gibt es kein Tool, was diese Symptome im Text zusammenfasst für den Arztbrief, … das Programm ist in der Lage, das zu kondensieren [und] gleichzeitig ein Anamnesetext für mich konzipiert und ich nur noch zwei, drei Sachen quasi ergänze oder ändere, dann wäre es auf jeden Fall extrem hilfreich. Also wir sitzen ja viel zu viel und dokumentieren auch unentwegt. Also ich meine, wir schreiben Medikamentenlisten ab“ (Ä4).

### Integration der Ergebnisse

Die zusammenführende Betrachtung beider Erhebungen zeigt sowohl konvergierende als auch divergierende Perspektiven hinsichtlich der Nutzung und Integration dieses DGL. Insgesamt wird deutlich, dass er zwar als innovatives Instrument wahrgenommen wird, seine begrenzte Einbindung jedoch weniger auf eine grundsätzliche Ablehnung zurückzuführen ist als vielmehr auf strukturelle, technische und kontextuelle Barrieren entlang des gesamten Versorgungspfades.

Ein zentraler konvergenter Befund beider Erhebungen betrifft die technischen Herausforderungen. Während Patient:innen primär mit Zugangsproblemen wie instabiler Internetverbindung oder fehlender technischer Ausstattung konfrontiert sind, sehen sich Ärzt:innen mit systeminternen Hürden konfrontiert, insbesondere dem erschwerten Zugriff. Diese technischen Barrieren behindern sowohl die initiale Nutzung durch Patient:innen als auch die Weiterverwendung der generierten Daten im klinischen Alltag.

Es zeigt sich eine Diskrepanz in der Nutzenbewertung des DGL: Patient:innen stehen der Anwendung grundsätzlich offen gegenüber und nutzen sie unter anderem zum besseren Verständnis ihrer Symptome, während Ärzt:innen den Mehrwert für die klinische Entscheidungsfindung und die Reduktion der Arbeitsbelastung als begrenzt einschätzen und den DGL primär als Unterstützung für Patient:innen sehen. Auch bei der Bewertung Verlässlichkeit der Daten bestehen Unterschiede: Ein Drittel der Patient:innen vertraut den Empfehlungen, wohingegen Ärzt:innen Bedenken hinsichtlich eines systematischen Upcoding sowie algorithmischer Absicherungstendenzen äußern.

Beide Perspektiven weisen jedoch auf Probleme bezüglich der Datengrundlage hin. Patient:innen kritisieren die eingeschränkten Antwortmöglichkeiten und die fehlende Flexibilität der Symptomerfassung, wodurch individuelle Beschwerden nur unzureichend abgebildet werden. Damit korrespondieren die Zweifel der Ärzt:innen an der Validität und Verlässlichkeit der generierten Informationen.

Zudem verdeutlichen die Ergebnisse, dass der Nutzungskontext der ZNA die Integration des DGL limitiert. Für Patient:innen erfolgt die Nutzung häufig situativ unter Zeitdruck und teilweise unter gesundheitlichen Belastungen. Gleichzeitig erleben Ärzt:innen den DGL als zusätzlichen Aufwand in einem ohnehin hoch verdichteten Arbeitsumfeld ohne erkennbare Zeitersparnis. Vor dem Hintergrund bestehender Doppelstrukturen, insbesondere durch etablierte Triage-Systeme, wird der DGL daher eher als redundant denn als entlastend wahrgenommen.

Trotz dieser Barrieren zeigen beide Perspektiven vorhandene Potenziale der Anwendung. Patient:innen nutzen den DGL zur ersten Orientierung, während Ärzt:innen Vorteile insbesondere in einer Strukturierung von Informationen, in der Organisation des Wartezimmers sowie in einer unterstützenden (Nach‑)Triagierung sehen. Auch die Überbrückung von Sprachbarrieren wurde positiv hervorgehoben.

## Diskussion

Insgesamt machen die Befragungsergebnisse deutlich, dass die erfolgreiche Implementierung des DGL weniger eine Frage der individuellen Akzeptanz als vielmehr der systemischen Passung ist. Erst durch die Reduktion technischer Hürden, eine verbesserte Informationsvermittlung, eine flexiblere Datenerhebung sowie die konsequente Integration in klinische Arbeitsabläufe kann das bestehende Potenzial ausgeschöpft werden.

Ein Großteil der Patient:innen kam erstmals in der ZNA mit der Anwendung in Berührung, was gegen eine etablierte Nutzung des DGL vor der Inanspruchnahme medizinischer Versorgungen spricht. Dies steht im Einklang mit den Ergebnissen einer Befragung aus dem Jahr 2023, der zufolge nur eine Minderheit der Bevölkerung Symptomchecker kannte [15].

Die festgestellten Unterschiede in der Bildungs- und Digitalkompetenz zwischen (Nicht‑)Nutzer:innen sind konsistent mit bisherigen Erkenntnissen, wonach Symptomchecker eher von Personen mit höheren Kompetenzen genutzt werden [15–17]. Dies birgt die Gefahr einer Verstärkung gesundheitlicher Ungleichheiten. Dem kann durch den Abbau technischer Zugangsbarrieren sowie durch niedrigschwellige Zugänge unabhängig von der individuellen technischen Ausstattung und einer stabilen Internetverbindung entgegengewirkt werden. Frei zugängliche Terminals, etwa in Form sogenannter digitaler Tresen, können dazu beitragen, Symptomchecker für alle verfügbar zu machen [18]. Ergänzend können alternative Eingabemöglichkeiten wie sprachbasierte Systeme die Nutzung erleichtern. Ziel sollte eine inklusive Gestaltung sein, die unterschiedliche Kompetenzniveaus berücksichtigt und eine gleichberechtigte Nutzung unabhängig vom Besitz eines eigenen Endgeräts ermöglicht. Darüber hinaus zeigten Nichtnutzer:innen eine höhere Bereitschaft zur Weiterempfehlung des DGL als Nutzer:innen. Im Sinne des UTAUT-Modells (Unified Theory of Acceptance and Use of Technology) könnte dies darauf zurückzuführen sein, dass Nichtnutzer:innen den erwarteten Nutzen stärker gewichten, während Nutzer:innen ihre Bewertung auf tatsächliche Nutzungserfahrungen stützen [13].

Die Ergebnisse zeigen eine grundsätzlich positive Haltung der Ärzt:innen gegenüber Symptomcheckern, bei kritischer Bewertung der konkreten Implementierung des DGL in der ZNA. Insbesondere die wahrgenommene Nützlichkeit, die Zuverlässigkeit des Systems und die Selbstangabe werden infrage gestellt. Dies wird durch aktuelle Evidenz gestützt, wonach Symptomchecker zwar subjektiv als hilfreich gelten, jedoch keine messbaren Verbesserungen in der Versorgungsqualität oder der Interaktion zwischen Ärzt:innen und Patient:innen erzielen [19]. Eine qualitative Studie zeigt zudem, dass Patient:innen Angaben auf Schmerzskalen mitunter strategisch an erwartete Konsequenzen anpassen, was die Komplexität der Interpretation von Selbstangaben verdeutlicht [20].

Die diagnostische Genauigkeit von Symptomcheckern gilt im Allgemeinen als gering und ist sowohl mit Über- als auch mit Untertriagierung verbunden [17, 21]. Die von mehreren Ärzt:innen beobachtete Tendenz des DGL zu einer höheren Triagierung deutet auf eine defensiv orientierte Systemgestaltung hin. Eine solche Risikoaversion von Symptomcheckern ist bereits dokumentiert [22]: Notfälle werden zuverlässig erkannt, zugleich kommt es jedoch häufig zu Übertriagierungen.

Die Befragung der Ärzt:innen verweist zudem auf das Problem von Fixierungsfehlern im Sinne eines Automation-Bias [23]. Wird zu früh auf die technisch generierten Ergebnisse vertraut, können Fehler übersehen werden und die Ergebnisse als Anker weitere Handlungspfade und -möglichkeiten einschränken. Um eine unkritische Übernahme möglicher Fehler zu vermeiden, sollte die Einführung von Systemen wie Symptomchecker mit entsprechenden Strategien zum Umgang mit automatisierten Empfehlungen verbunden werden. Dazu zählen Ansätze des Cognitive Debiasing [24], die dazu anregen, vom Problem zurückzutreten und bisherige Einschätzungen und Entscheidungsschritte kritisch zu hinterfragen.

In der aktuellen Ausgestaltung sehen die Ärzt:innen die Anwendung eher als Tool zur Organisation des Wartezimmers. Eine Erweiterung um die digitale Selbstanamnese der Patient:innen sowie eine umfassende Anbindung an das KIS könnten die Dokumentationsarbeit der Ärzt:innen potenziell reduzieren, während eine Verbesserung der diagnostischen Genauigkeit bislang nicht nachgewiesen ist [25].

Das hier untersuchte Instrument ist eines von mehreren Systemen zur Patient:innensteuerung; vergleichbar ist unter anderem der Patientennavigator der 116117 [9]. Auch wenn beide auf die gleiche Software zur Ersteinschätzung zurückgreifen, bietet der DGL durch die Möglichkeit der direkten Einbindung in Versorgungsstrukturen der ZNA einen zusätzlichen Vorteil, der über die reine Auskunft hinausgeht und bei angemessener Implementierung die Behandlung der Patient:innen unterstützen kann. Die bestehenden Defizite in der Prozessintegration, insbesondere die erschwerte Auffindbarkeit und redundante Arbeitsschritte im KIS, verdeutlichen den Bedarf an einer nahtlosen technischen Integration. Standardisierte Schnittstellen können die Dokumentationslast reduzieren und die Akzeptanz digitaler Assistenzsysteme fördern.

Die Ergebnisse unterliegen mehreren Limitationen, darunter die Durchführung an einem einzelnen Studienzentrum, eine kleine Stichprobe, ein teilweise hoher Anteil fehlender Angaben (vgl. Items zu Vertrauen, Nützlichkeit) sowie der explorative Charakter der Daten. Diese Faktoren schränken die Übertragbarkeit und Aussagekraft der Ergebnisse ein.

## Fazit

Symptomchecker wie dieser DGL werden grundsätzlich akzeptiert und bieten Potenziale für die Notfallversorgung. Die Nutzung hängt jedoch von einer besseren systemischen Integration, einer höheren Verlässlichkeit der Daten sowie einem niedrigschwelligen und inklusiven Zugang ab. Zukünftige Forschung sollte daher insbesondere die Integration in klinische Arbeitsabläufe, eine inklusive Ausgestaltung der Nutzung sowie die Effekte auf die Versorgungsqualität und die Potenziale für die Patient:innensteuerung systematisch untersuchen.

## Data Availability

Die während der vorliegenden Studie erzeugten und/oder analysierten Datensätze sind auf begründete Anfrage bei der Korrespondenzperson erhältlich.
